# Supplementary material for: Tcf1+ cells are required to maintain the inflationary T cell pool upon MCMV infection
Source: Nat Commun. 2020 May 8;11:2295. doi: 10.1038/s41467-020-16219-3 (PMC7211020; doi:10.1038/s41467-020-16219-3)
Supplement: Supplementary file 1 — Supplementary Information [file 41467_2020_16219_MOESM1_ESM.pdf]

Supplementary information belonging to

Tcf1$^+$ cells are required to maintain the inflationary T cell pool upon MCMV infection

Welten et al.

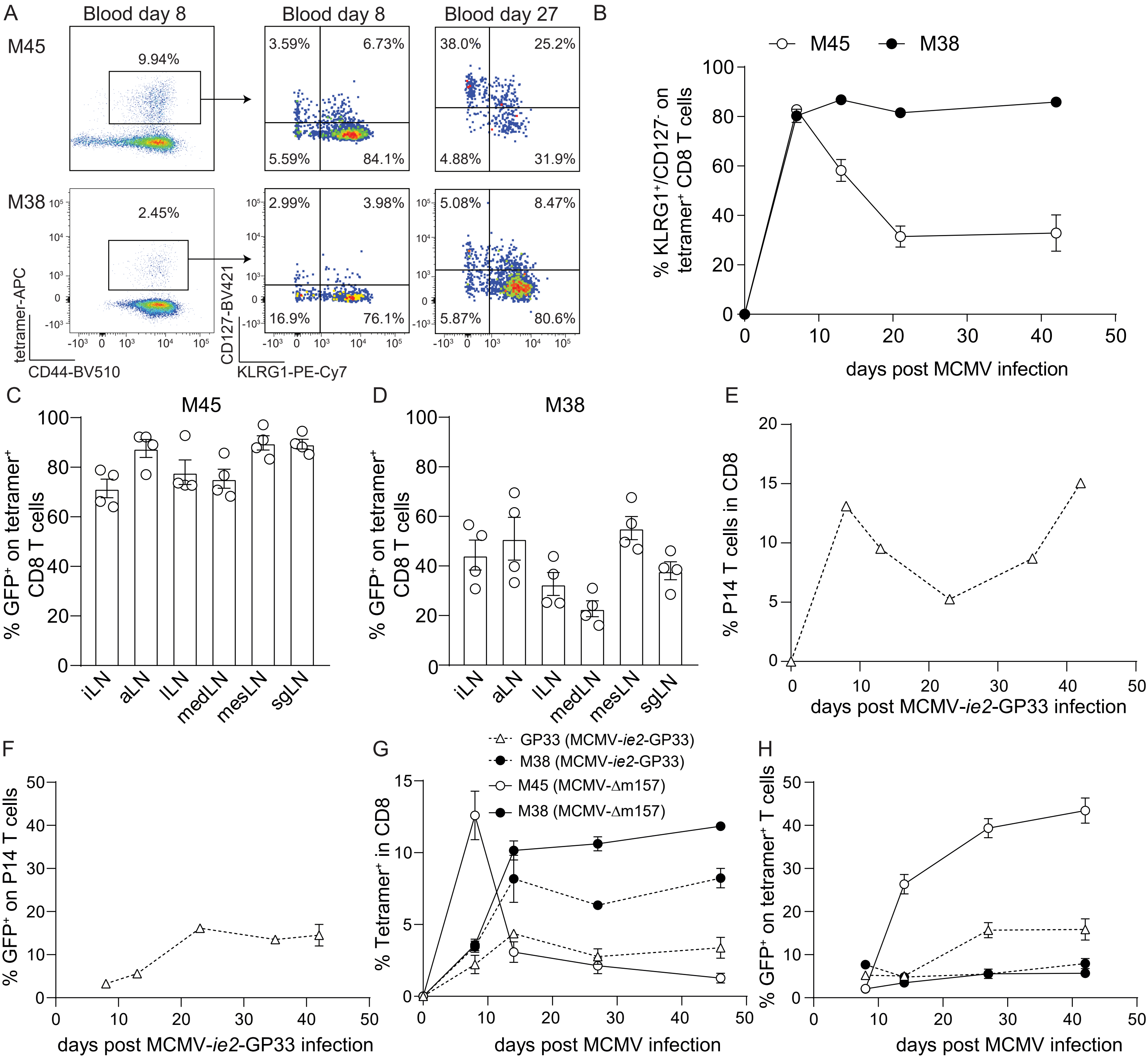

Supplementary Fig. 1: Inflationary T cells have a predominant effector memory phenotype.

Tcf7^{GFP} mice were infected i.v. with 10^6 PFU MCMVΔm157. (A) At day 8 post-infection, the blood of MCMV infected mice was assessed for binding to MHC Class I tetramers and CD44 expression. Cells were pre-gated on CD8 T cells. The expression of CD127 and KLRG1 is shown on M45- and M38-specific CD8 T cells on day 8 and day 27 post-infection in the blood. (B) The percentage of MCMV-specific CD8 T cells that expresses KLRG1 is shown in the blood as mean ± SEM (n=4 mice) of 1 experiment out of 3 independent experiments. (C+D) The percentage of M45- (C) and M38-specific (D) cells that expresses GFP in the inguinal- (iLN), axillary- (aLN), lumbar- (lLN), mediastinal- (medLN), mesenteric- (mesLN) and salivary gland-LN (sgLN) is shown on day 50 post-infection. Bar graphs represent mean ± SEM (n=4). (E) 1 × 10^5 CD45.1$^+$ *Tcf7*^{GFP} P14 T cells were adoptively transferred into CD45.2$^+$ hosts that were subsequently infected with 2 × 10^5 PFU MCMV-*ie2*-GP33. The percentage of P14 T cells in CD8 T cells was longitudinally tracked in the blood (n=2). (F) The percentage of P14 T cells that expresses GFP is shown in the blood as mean ± SEM (n=2-6). (G) *Tcf7*^{GFP} mice were i.v. infected with 2 × 10^5 PFU MCMV-*ie2*-GP33 (expressing m157) or 10^6 PFU MCMV-Δm157. The percentage of MHC class I tetramer binding cells is shown in the blood as mean ± SEM, (GP33 and M38 for MCMV-*ie2*-GP33 infection, M45 and M38 for MCMVΔm157) (n=3-4). (H) The percentage GFP expressing cells within the tetramer population is shown as mean ± SEM of the mice described in E (n=2-4). Data are representative of 1 out of 2 independent experiments with 2-6 mice per group. Source data are provided as a Source Data File.

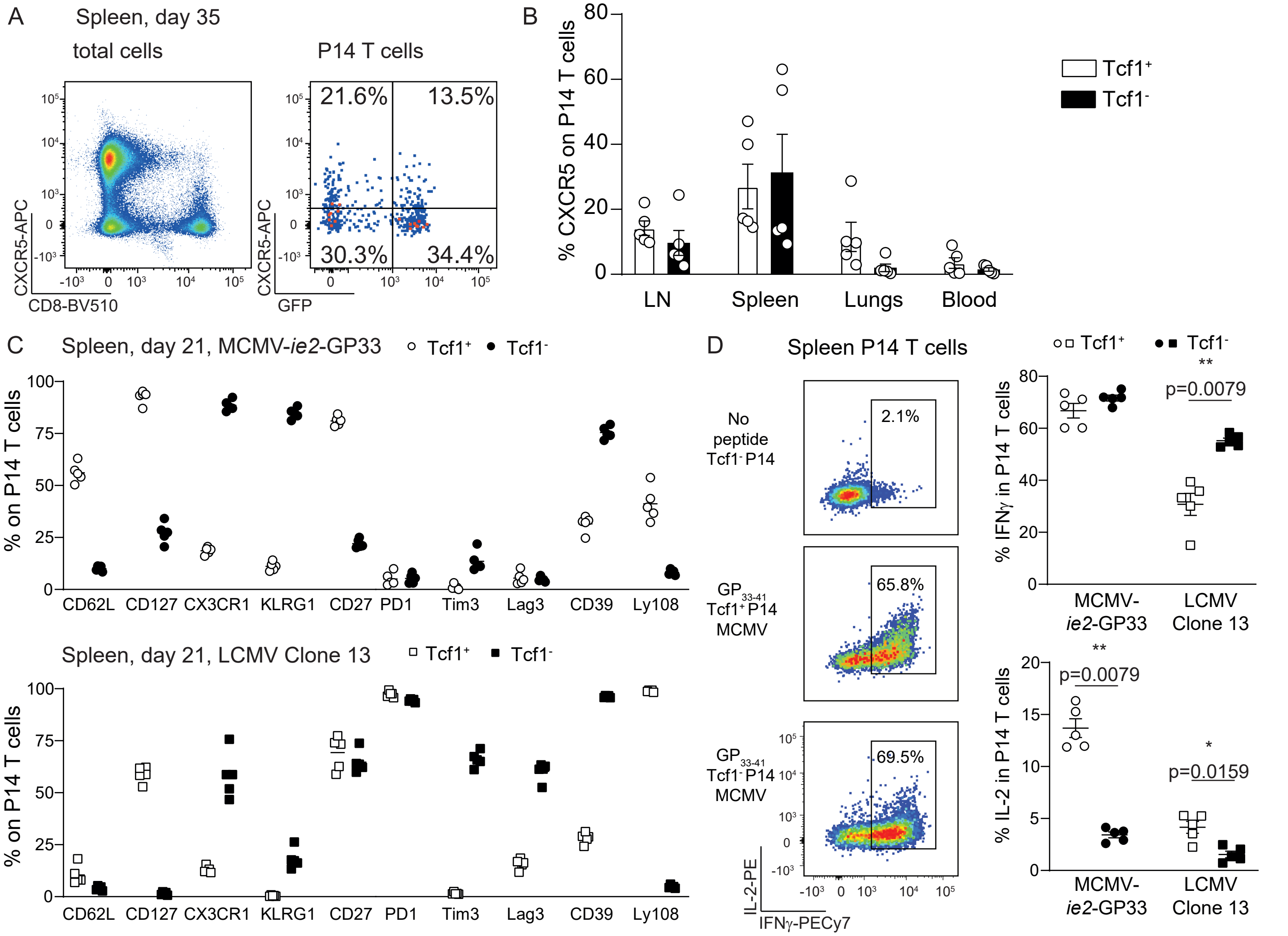

Supplementary Fig. 2: Phenotypic characterization of Tcf1⁺ cells in MCMV and LCMV infection. CD45.1⁺ *Tcf7*^{GFP} P14 cells were transferred into WT mice that were subsequently infected with 2 × 10^5 PFU MCMV-*ie2*-GP33. (A) Flow cytometry plots show the expression of CXCR5 versus CD8 on all cells (left) and versus GFP (Tcf1) on P14 cells in the spleen (right). (B) Bar graphs show the percentage of Tcf1$^+$ (GFP$^+$) and Tcf1$^-$ (GFP$^-$) P14 cells expressing CXCR5 as mean ± SEM, each dot represents an individual mouse. Data are representative of 1 out of 2 experiments (n=5). (C) 10^4 CD45.1$^+$ *Tcf7*^{GFP} P14 cells were transferred into WT mice that were subsequently infected with 2 × 10^5 PFU MCMV-*ie2*-GP33 or with 2 × 10^6 ffu LCMV-Clone 13. The expression of several cell surface markers is shown on splenic Tcf1$^+$ and Tcf1$^-$ P14 cells, 21 days post-infection. Mean is indicated (n=5). (D) Tcf1$^+$ and Tcf1$^-$ P14 cells were sorted from the spleen of MCMV- and LCMV-infected mice (as in C). Shown are flow cytometry plots for the intracellular IFNγ and IL-2 production after restimulation with $GP_{33\text{-}41}$ peptide and the percentage of P14 cells that produces IFNγ or IL-2 as mean ± SEM. Data are from 1 experiment, each dot is an individual mouse (n=5). *p<0.05, **p<0.01; statistical significance was determined using two-sided non-parametric Mann-Whitney test. Source data are provided as a Source Data File.

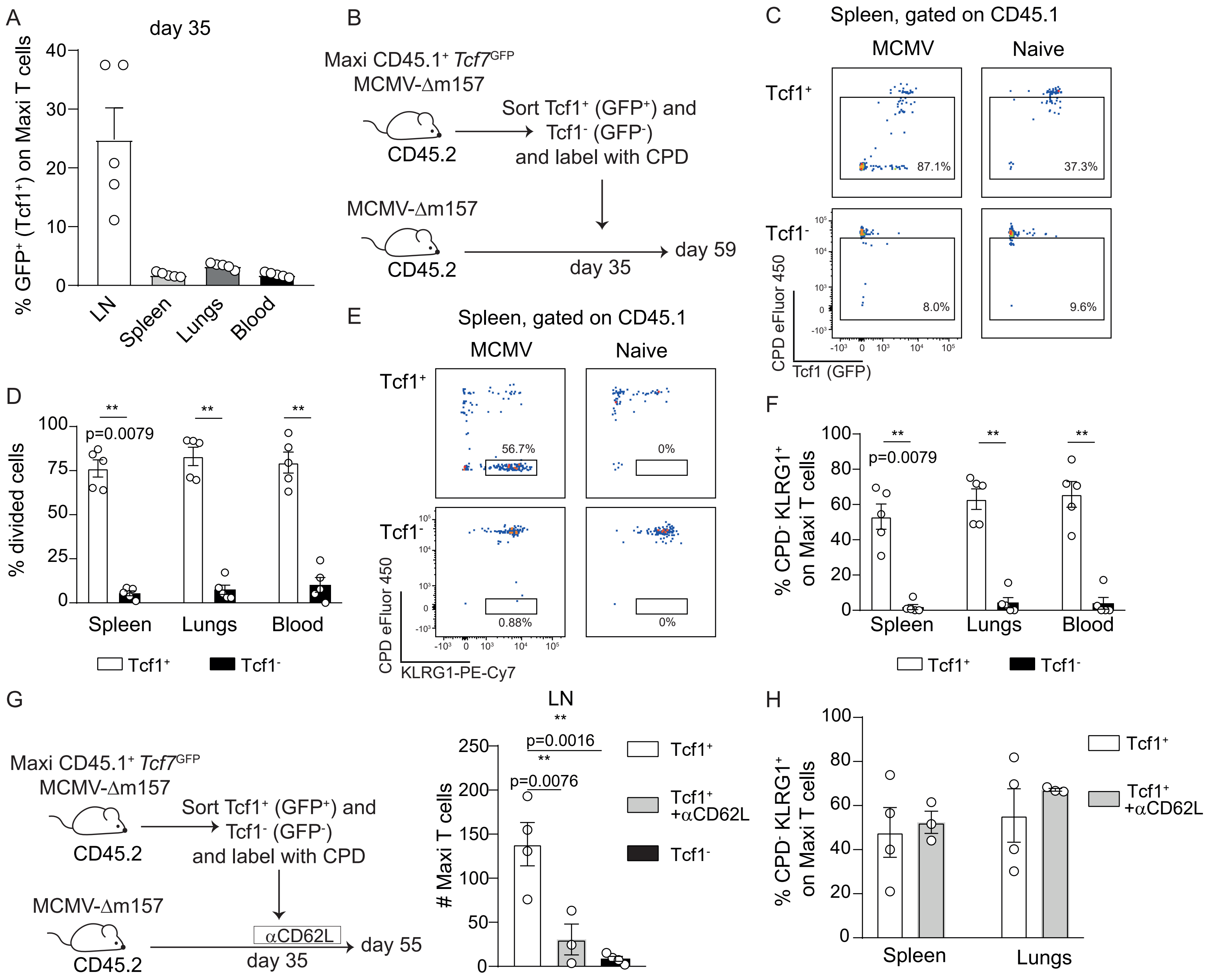

Supplementary Fig. 3: Tcf1$^+$ Maxi cells give rise to Tcf1$^-$ KLRG1$^+$ cells upon MCMV infection.

CD45.1$^+$ *Tcf7*^{GFP} Maxi cells were transferred into CD45.2$^+$ hosts that were subsequently i.v. infected with 10^6 PFU MCMV Δm157. (A) Percentage of Maxi cells that expresses Tcf1 (GFP) is indicated as mean + SEM (n=5). (B) Experimental setup: 35 days post-transfer of *Tcf7*^{GFP} Maxi cells, Tcf1$^+$ and Tcf1$^-$ Maxi cells were sorted from MCMV infected mice and labelled with CPD. 2 × 10^4 Tcf1$^+$ or Tcf1$^-$ Maxi cells were transferred in infection-matched recipients. After 24 days the proliferation history of Maxi cells was determined. Naive hosts received 1 × 10^5 Maxi cells. (C) Representative flow cytometry plot shows CPD dilution versus Tcf1 expression (GFP), on Maxi cells in the spleen. (D) Percentage of Maxi cells that divided at least once is shown (n=5). (E) Representative flow cytometry plot shows the proliferation history of Maxi cells based on CPD dilution versus KLRG1 expression in the spleen. (F) Percentage of Maxi T cells that has out-diluted CPD and expresses KLRG1 is shown (n=5). (G) Experimental setup: CD45.1$^+$ *Tcf7*^{GFP} Maxi cells were transferred into CD45.2$^+$ hosts that were subsequently i.v. infected with 10^6 PFU MCMVΔm157. 35 days post-infection, Tcf1$^+$ Maxi cells were sorted from MCMV infected mice, and labelled with CPD. 5 × 10^4 Tcf1$^+$ Maxi cells were adoptively transferred into infection-matched recipients. One group of mice received αCD62L-blocking antibodies starting 8 hours before Maxi transfer. 20 days post Maxi transfer the proliferation history of Maxi cells was determined in the new hosts. Bar graph show the total number of Maxi cells in the inguinal LN after transfer of Tcf1$^+$ or Tcf1$^-$ Maxi cells (n=3-4). (H) Percentage of Maxi cells that has out-diluted the proliferation dye and expresses KLRG1 is shown (n=3-4). Bar graphs (D-H) represent mean ± SEM, each dot represents an individual mouse. Data show 1 out of 3 independent experiments with 3-5 mice per group. **$p<0.01$; statistical significance was determined using the two-sided non-parametric Mann-Whitney test or the one-way ANOVA using Tukey's multiple comparisons test (G). Source data are provided as a Source Data File.

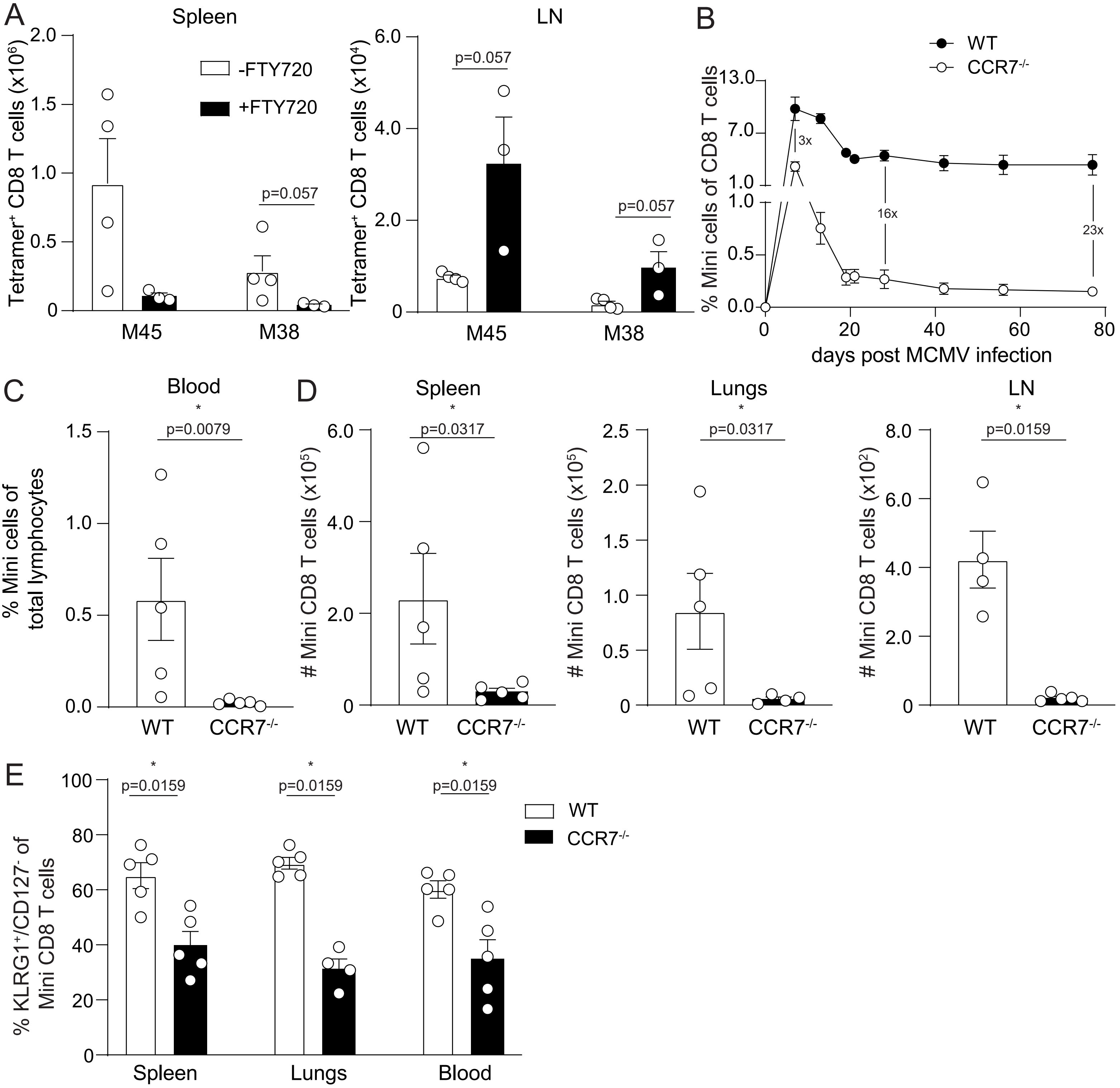

Supplementary Fig. 4: CCR7 deficiency leads to a diminished inflationary T cell response.

(A) Mice were infected i.v. with 10^6 PFU MCMV-Δm157. One group of mice received 5 µg/ml FTY720 in the drinking water throughout the experiment starting one day prior to infection. On day 7 post-infection, the MCMV-specific CD8 T cell response was determined by MHC class I tetramer binding in the spleen (left) and the LNs (right). Bar graphs show mean + SEM. Data of 1 experiment is shown (n=3-4). (B-D) Experimental setup: 2×10^5 naïve $CD45.1^+$ WT or $CCR7^{-/-}$ Mini cells were adoptively transferred into $CD45.2^+$ hosts prior to an i.v. infection with 5×10^6 PFU MCMV-Δm157 infection. (B) Percentages of Mini cells in the blood is shown as mean ± SEM (n=5). (C) The percentage of WT and $CCR7^{-/-}$ Mini cells amongst total lymphocytes in the blood is shown at day 77 post-infection as mean ± SEM (n=5). (D) Total numbers of WT and $CCR7^{-/-}$ Mini cells in the lung, spleen and inguinal LN at day 83 post-infection are shown as mean ± SEM (n=4-5). (E) Percentages of $KLRG1^+$ $CD127^-$ Mini cells are shown. The spleen and the lungs represent day 83 post-infection and the blood is shown for day 77 post-infection. Bar graphs show mean ± SEM of 1 experiment (n=4-5). (A-E) *$p<0.05$; statistical analyses were performed using the two-sided non-parametric Mann-Whitney U test. Source data are provided as a Source Data File.

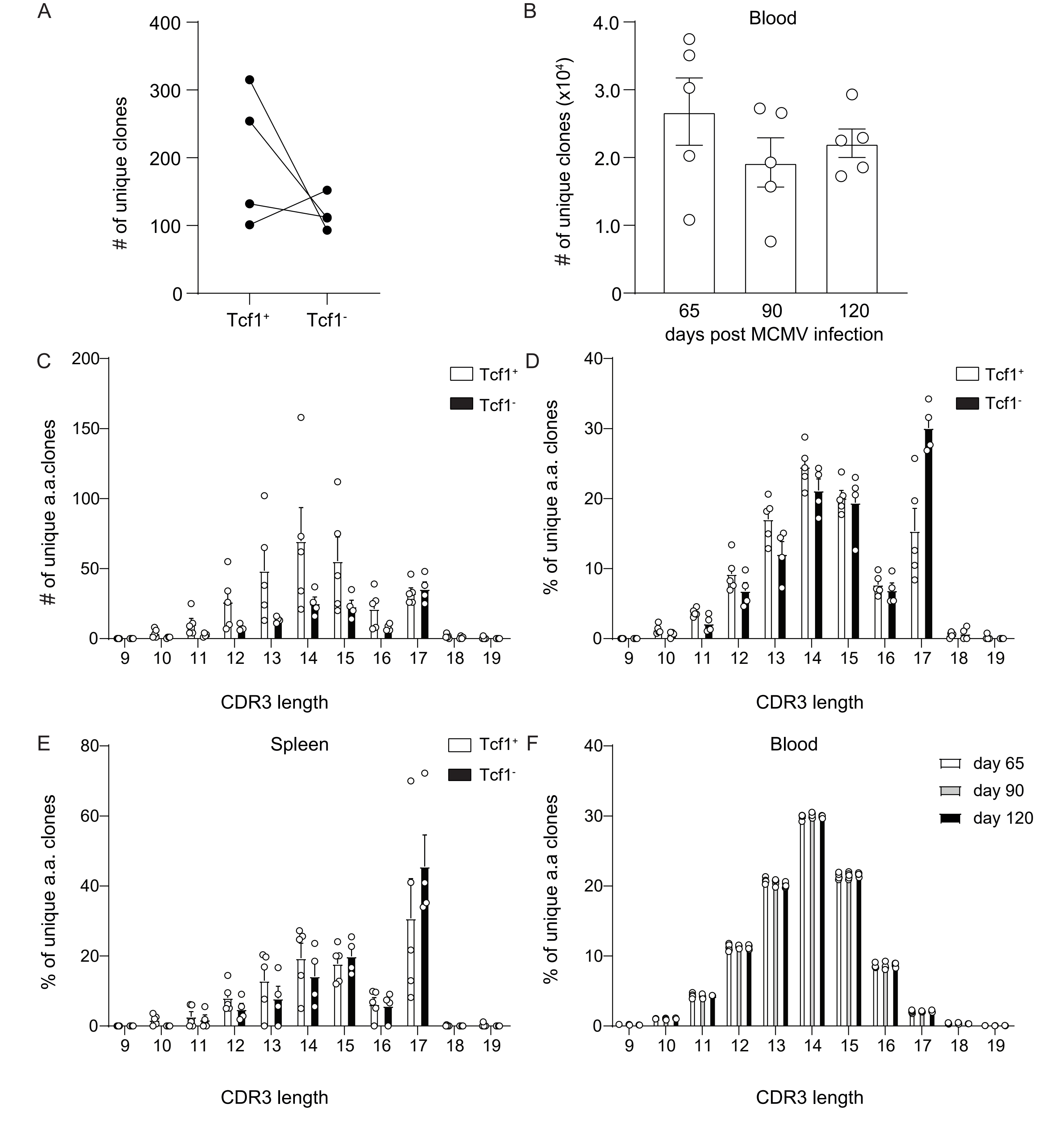

Supplementary Fig. 5: The CDR3 length distribution amongst Tcf1⁺ and Tcf1⁻ M38-specific CD8 T cells.

(A) The total number of clones, determined by unique amino acid sequence of the CDR3 are shown for the Tcf1⁺ and Tcf1⁻ M38-specific CD8 T cells pooled from the lungs, spleen and LNs. Each line represents an individual mouse and lines connect data from the same mouse (n=4). (B) The total number of unique amino acid clones are shown in the blood for day 65, 90 and 120 post MCMV infection as mean ± SEM. Each dot represents an individual mouse (n=5). (C) The total number of unique clones found for each CDR3 length is shown for the total Tcf1⁺ and Tcf1⁻ M38-specific CD8 T cells pooled from the lungs, spleen and LNs (n=4-5). (D) Distribution of the CDR3 length selection is shown for the total number of unique amino acid clones within the Tcf1⁺ and Tcf1⁻ M38-specific CD8 T cells pooled from the lungs, spleen and LNs (n=4-5). (E) Distribution of the CDR3 length selection is shown for the total number of unique amino acid clones within the Tcf1⁺ and Tcf1⁻ M38-specific CD8 T cells found in the spleen (n=4-5). (F) Distribution of the CDR3 length selection of the unique amino acid clones found in the blood at day 65, 90 and day 120 post MCMV infection is shown (n=5). All bar graphs represent mean + SEM, pooled from n=4-5 mice of 1 experiment. Source data are provided as a Source Data File.

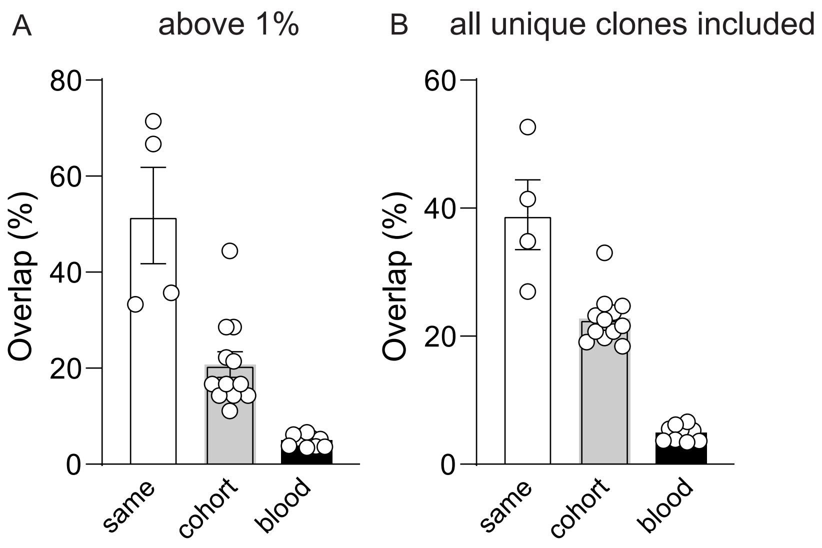

Supplementary Fig. 6: Clonal overlap between the Tcf1⁻ and Tcf1⁺ compartment.

Bar graphs show the percentage of $Tcf1^-$ clones that is found within the $Tcf1^+$ compartment compared to the same mouse or compared to a different mouse. (A) All unique clones are included that are found above 1% in the repertoire. (B) All unique clones found in each mouse are included. Blood indicates the percentage of overlap of all unique clones between different mice at day 90 post-infection. All bar graphs represent mean ± SEM, (n=4). Source data are provided as a Source Data File.

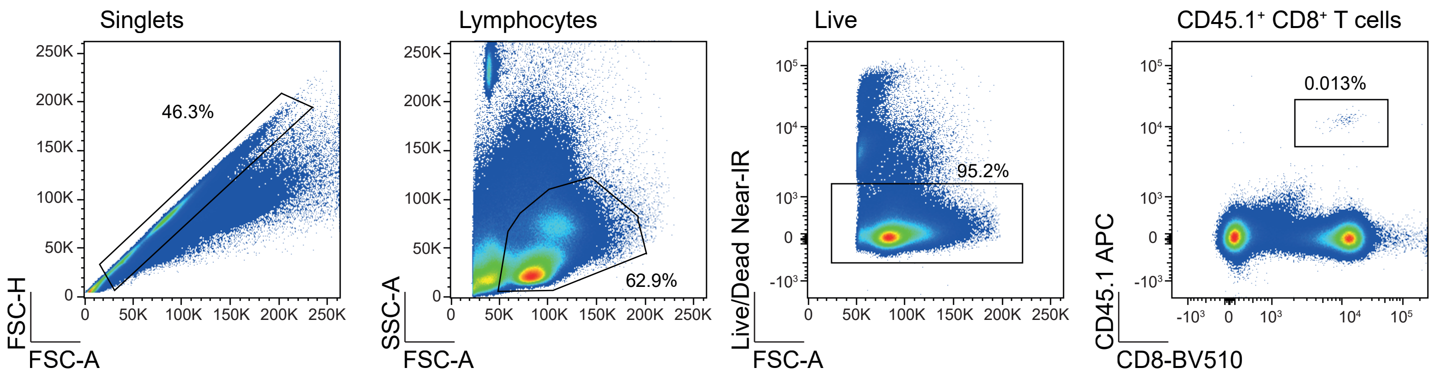

Supplementary Fig. 7: Flow cytometry gating strategy.

For all samples the following gating strategy was used, exclusion of doublets (FSC-H/FSC-A), lymphocytes (SSC-A/FSC-A), live cells (Live/Dead marker Near-IR/FSC-A). To identify congenically marked TCR transgenic CD8 T cells, cells were gated for CD8 and for the congenic marker.

Marker	Fluorochrome	Manufacturer	Catalag number	Clone	Dilution
B220	Biotin	Biolegend	103204	RA3-6B2	1/100
B220	APC	BioLegend	103212	RA3-6B	1/100
CD4	Biotin	Biolegend	100404	GK1.5	1/100
CD8	BV421	BioLegend	100738	53-6.7	1/100
CD8	Percp	BioLegend	100732	53-6.7	1/200
CD8	BV510	BioLegend	100752	53-6.7	1/500
CD8	BV605	Biolegend	100744	53.6.7	1/500
CD8	BV570	BioLegend	100739	53-6.7	1/200
CD27	BV421	Biolegend	124223	LG.3A10	1/200
CD39	AlexaFluor 647	BioLegend	143807	Duha59	1/200
CD44	PE	BioLegend	103008	IM7	1/500
CD45.1	PE	BioLegend	110708	A20	1/200
CD45.1	APC	BioLegend	110714	A20	1/200
CD45.1	BV421	BioLegend	110731	A20	1/400
CD45.1	PB	BioLegend	110722	A20	1/200
CD45.1	BV510	BioLegend	110741	A20	1/200
CD45.1	BV711	Biolegend	110739	A20	1/300
CD45.2	Percp	BioLegend	109825	104	1/200
CD62L	Percp	BioLegend	104430	MEL-14	1/200
CD62L	BUV395	BD	740218	MEL-14	1/200
CD90.1	APC	eBioscience	17-0900-82	HIS51	1/1000
CD127	BV421	BioLegend	135023	A7R34	1/100
CD169	AF647	BioLegend	142408	3D6.112	1/200
CX3CR1	APC	Biolegend	149007	SA011F11	1/200
CXCR5	Biotin	Biolegend	145510	L138D7	1/100
IFNy	PECy7	Biolegend	505826	XMG1.2	1/200
IgM	BV421	BioLegend	406517	RMM-1	1/100
IL-2	PE	BioLegend	503808	JES6-5H4	1/100
KLRG1	PECy7	eBioscience	25-5893-82	2F1	1/200
KLRG1	BUV395	BD	740279	2F1	1/200
Lag3	PE	BD	552380	C9B7W	1/200
Ly108	APC	BioLegend	134609	330-AJ	1/200
PD-1	PECy7	Biolegend	135215	29F.1A12	1/200
Streptavidin	APC	Biolegend	405207		1/500
Tcf1	PE	Cell signaling	14456S	C63D9	1/200
Tim-3	PE	Biolegend	119703	RMT3-23	1/200
Va2	PE	BioLegend	127808	B20.1	1/500
CD62L	n.a.	B-Xcell	BE0021	MEL-14	

Supplementary Table 1: Antibodies. Information regarding the antibodies used in this study is summarized.

Name	Primer sequence (5’→3’)
**PCR 1**	
TRBV1	CCCTCCTTTAATTCCCTACCACGTGGTCAAGCTG
TRBV2	CCCTCCTTTAATTCCCCAGTATCTAGGCCACAATGC
TRBV3	CCCTCCTTTAATTCCCCCCAAAGTCTTACAGATCCC
TRBV4	CCCTCCTTTAATTCCCGACGGCTGTTTTCCAGAC
TRBV5	CCCTCCTTTAATTCCCGGTATAAACAGAGCGCTGAG
TRBV12	CCCTCCTTTAATTCCCGGGGTTGTCCAGTCTCC
TRBV13	CCCTCCTTTAATTCCCGCTGCAGTCACCCAAAG
TRBV14	CCCTCCTTTAATTCCCGCAGTCCTACAGGAAGGG
TRBV15	CCCTCCTTTAATTCCCGAGTTACCCAGACACCCAG
TRBV16	CCCTCCTTTAATTCCCCCTAGGCACAAGGTGACAG
TRBV17	CCCTCCTTTAATTCCCGAAGCCAAACCAAGCAC
TRBV19	CCCTCCTTTAATTCCCGATTGGTCAGGAAGGGC
TRBV20	CCCTCCTTTAATTCCCGGATGGAGTGTCAAGCTG
TRBV23	CCCTCCTTTAATTCCCCTGCAGTTACACAGAAGCC
TRBV24	CCCTCCTTTAATTCCCCAGACTCCACGATACCTGG
TRBV26	CCCTCCTTTAATTCCCGGTGAAAGGGCAAGGAC
TRBV31	CCCTCCTTTAATTCCCCTAACCTCTACTGGTACTGGCAG
TRBV29	CCCTCCTTTAATTCCCGCTGGAATGTGGACAGG
TRBV30	CCCTCCTTTAATTCCCCCTCCTCTACCAAAAGCC
TCR internal	GAG GAG AGA GAG AGA GGG GTA GCC TTT TGT TTG TTT G
**PCR 2**	
Univ-Fw	AAT GAT ACG GCG ACC ACC GAG ATC TAC ACT CTT TCC CTA CAC GAC GCT CTT CCG ATC TNN NNC CCT CCT TTA ATT CCC
Idx1-Rv	CAA GCA GAA GAC GGC ATA CGA GAT CGT GAT GTG ACT GGA GTT CAG ACG TGT GCT CTT CCG ATC TNN NNG AGG AGA GAG AGA GAG
Idx2-Rv	CAA GCA GAA GAC GGC ATA CGA GAT ACA TCG GTG ACT GGA GTT CAG ACG TGT GCT CTT CCG ATC TNN NNG AGG AGA GAG AGA GAG
Idx3-Rv	CAA GCA GAA GAC GGC ATA CGA GAT GCC TAA GTG ACT GGA GTT CAG ACG TGT GCT CTT CCG ATC TNN NNG AGG AGA GAG AGA GAG
Idx4-Rv	CAA GCA GAA GAC GGC ATA CGA GAT TGGTCA GTG ACT GGA GTT CAG ACG TGT GCT CTT CCG ATC TNN NNG AGG AGA GAG AGA GAG
Idx5-Rv	CAA GCA GAA GAC GGC ATA CGA GAT CACTGT GTG ACT GGA GTT CAG ACG TGT GCT CTT CCG ATC TNN NNG AGG AGA GAG AGA GAG
Idx6-Rv	CAA GCA GAA GAC GGC ATA CGA GAT ATTGGC GTG ACT GGA GTT CAG ACG TGT GCT CTT CCG ATC TNN NNG AGG AGA GAG AGA GAG
Idx7-Rv	CAA GCA GAA GAC GGC ATA CGA GAT GATCTG GTG ACT GGA GTT CAG ACG TGT GCT CTT CCG ATC TNN NNG AGG AGA GAG AGA GAG

Idx8-Rv	CAA GCA GAA GAC GGC ATA CGA GAT TCAAGT GTG ACT GGA GTT CAG ACG TGT GCT CTT CCG ATC TNN NNG AGG AGA GAG AGA GAG
Idx9-Rv	CAA GCA GAA GAC GGC ATA CGA GAT CTGATC GTG ACT GGA GTT CAG ACG TGT GCT CTT CCG ATC TNN NNG AGG AGA GAG AGA GAG
Idx10-Rv	CAA GCA GAA GAC GGC ATA CGA GAT AAGCTA GTG ACT GGA GTT CAG ACG TGT GCT CTT CCG ATC TNN NNG AGG AGA GAG AGA GAG
Idx11-Rv	CAA GCA GAA GAC GGC ATA CGA GAT GTAGCC GTG ACT GGA GTT CAG ACG TGT GCT CTT CCG ATC TNN NNG AGG AGA GAG AGA GAG
Idx12-Rv	CAA GCA GAA GAC GGC ATA CGA GAT TACAAG GTG ACT GGA GTT CAG ACG TGT GCT CTT CCG ATC TNN NNG AGG AGA GAG AGA GAG
Idx13-Rv	CAA GCA GAA GAC GGC ATA CGA GAT TGTTGACT GTG ACT GGA GTT CAG ACG TGT GCT CTT CCG ATC TNN NNG AGG AGA GAG AGA GAG
Idx14-Rv	CAA GCA GAA GAC GGC ATA CGA GAT ACGGAACT GTG ACT GGA GTT CAG ACG TGT GCT CTT CCG ATC TNN NNG AGG AGA GAG AGA GAG
Idx15-Rv	CAA GCA GAA GAC GGC ATA CGA GAT TCTGACAT GTG ACT GGA GTT CAG ACG TGT GCT CTT CCG ATCTNN NNG AGG AGA GAG AGA GAG
Idx16 rev	CAA GCA GAA GAC GGC ATA CGA GAT CGGGACGG GTG ACT GGA GTT CAG ACG TGT GCT CTT CCG ATC TNN NNG AGG AGA GAG AGA GAG
Idx17 rev	CAA GCA GAA GAC GGC ATA CGA GAT CTCTAC GTG ACT GGA GTT CAG ACG TGT GCT CTT CCG ATC TNN NNG AGG AGA GAG AGA GAG
Idx18 rev	CAA GCA GAA GAC GGC ATA CGA GAT GTGCGGAC GTG ACT GGA GTT CAG ACG TGT GCT CTT CCG ATC TNN NNG AGG AGA GAG AGA GAG
Idx19 rev	CAA GCA GAA GAC GGC ATA CGA GAT CGTTTCAC GTG ACT GGA GTT CAG ACG TGT GCT CTT CCG ATC TNN NNG AGG AGA GAG AGA GAG
Idx20 rev	CAA GCA GAA GAC GGC ATA CGA GAT AAGGCCAC GTG ACT GGA GTT CAG ACG TGT GCT CTT CCG ATC TNN NNG AGG AGA GAG AGA GAG
Idx21 rev	CAA GCA GAA GAC GGC ATA CGA GAT TCCGAAAC GTG ACT GGA GTT CAG ACG TGT GCT CTT CCG ATC TNN NNG AGG AGA GAG AGA GAG
Idx22 rev	CAA GCA GAA GAC GGC ATA CGA GAT CGTACG GTG ACT GGA GTT CAG ACG TGT GCT CTT CCG ATCTNN NNG AGG AGA GAG AGA GAG
Idx23 rev	CAA GCA GAA GAC GGC ATA CGA GAT CCACTC GTG ACT GGA GTT CAG ACG TGT GCT CTT CCG ATC TNN NNG AGG AGA GAG AGA GAG
Idx24 rev	CAA GCA GAA GAC GGC ATA CGA GAT GCTACC GTG ACT GGA GTT CAG ACG TGT GCT CTT CCG ATC TNN NNG AGG AGA GAG AGA GAG
Idx25 rev	CAA GCA GAA GAC GGC ATA CGA GAT ATCAGT GTG ACT GGA GTT CAG ACG TGT GCT CTT CCG ATCTNN NNG AGG AGA GAG AGA GAG
Idx26 rev	CAA GCA GAA GAC GGC ATA CGA GAT GCTCAT GTG ACT GGA GTT CAG ACG TGT GCT CTT CCG ATC TNN NNG AGG AGA GAG AGA GAG
Idx27 rev	CAA GCA GAA GAC GGC ATA CGA GAT AGGAAT GTG ACT GGA GTT CAG ACG TGT GCT CTT CCG ATC TNN NNG AGG AGA GAG AGA GAG
Idx28 rev	CAA GCA GAA GAC GGC ATA CGA GAT CTTTTG GTG ACT GGA GTT CAG ACG TGT GCT CTT CCG ATC TNN NNG AGG AGA GAG AGA GAG
Idx29 rev	CAA GCA GAA GAC GGC ATA CGA GAT TAGTTG GTG ACT GGA GTT CAG ACG TGT GCT CTT CCG ATC TNN NNG AGG AGA GAG AGA GAG
Idx30 rev	CAA GCA GAA GAC GGC ATA CGA GAT CCGGTG GTG ACT GGA GTT CAG ACG TGT GCT CTT CCG ATC TNN NNG AGG AGA GAG AGA GAG
Idx31 rev	CAA GCA GAA GAC GGC ATA CGA GAT ATCGTG GTG ACT GGA GTT CAG ACG TGT GCT CTT CCG ATCTNN NNG AGG AGA GAG AGA GAG

Idx32 rev	CAA GCA GAA GAC GGC ATA CGA GAT TGAGTG GTG ACT GGA GTT CAG ACG TGT GCT CTT CCG ATC TNN NNG AGG AGA GAG AGA GAG
Idx33 rev	CAA GCA GAA GAC GGC ATA CGA GAT CGCCTG GTG ACT GGA GTT CAG ACG TGT GCT CTT CCG ATC TNN NNG AGG AGA GAG AGA GAG
Idx34 rev	CAA GCA GAA GAC GGC ATA CGA GAT GCCATG GTG ACT GGA GTT CAG ACG TGT GCT CTT CCG ATC TNN NNG AGG AGA GAG AGA GAG
Idx35 rev	CAA GCA GAA GAC GGC ATA CGA GAT AAAATG GTG ACT GGA GTT CAG ACG TGT GCT CTT CCG ATCTNN NNG AGG AGA GAG AGA GAG
Idx36 rev	CAA GCA GAA GAC GGC ATA CGA GAT TGTTGG GTG ACT GGA GTT CAG ACG TGT GCT CTT CCG ATC TNN NNG AGG AGA GAG AGA GAG
Idx37 rev	CAA GCA GAA GAC GGC ATA CGA GAT ATTCCG GTG ACT GGA GTT CAG ACG TGT GCT CTT CCG ATC TNN NNG AGG AGA GAG AGA GAG
Idx38 rev	CAA GCA GAA GAC GGC ATA CGA GAT AGCTAG GTG ACT GGA GTT CAG ACG TGT GCT CTT CCG ATC TNN NNG AGG AGA GAG AGA GAG
Idx39 rev	CAA GCA GAA GAC GGC ATA CGA GAT GTATAG GTG ACT GGA GTT CAG ACG TGT GCT CTT CCG ATC TNN NNG AGG AGA GAG AGA GAG
Idx40 rev	CAA GCA GAA GAC GGC ATA CGA GAT TCTGAG GTG ACT GGA GTT CAG ACG TGT GCT CTT CCG ATC TNN NNG AGG AGA GAG AGA GAG

Supplementary Table 2: Primers. Primers used throughout this study are indicated.
