# Supplementary material for: Tcf1+ cells are required to maintain the inflationary T cell pool upon MCMV infection
Source: Nat Commun. 2020 May 8;11:2295. doi: 10.1038/s41467-020-16219-3 (PMC7211020; doi:10.1038/s41467-020-16219-3)
Supplement: Supplementary file 3 — Reporting Summary [file 41467_2020_16219_MOESM3_ESM.pdf]

nature research

Corresponding author(s): Annette Oxenius

Last updated by author(s): Mar 22, 2020

Reporting Summary

Nature Research wishes to improve the reproducibility of the work that we publish. This form provides structure for consistency and transparency in reporting. For further information on Nature Research policies, see Authors & Referees and the Editorial Policy Checklist .

Statistics

For all statistical analyses, confirm that the following items are present in the figure legend, table legend, main text, or Methods section.

n/a	Confirmed	
☐	☒	The exact sample size (*n*) for each experimental group/condition, given as a discrete number and unit of measurement
☐	☒	A statement on whether measurements were taken from distinct samples or whether the same sample was measured repeatedly
☐	☒	The statistical test(s) used AND whether they are one- or two-sided<br>*Only common tests should be described solely by name; describe more complex techniques in the Methods section.*
☒	☐	A description of all covariates tested
☐	☒	A description of any assumptions or corrections, such as tests of normality and adjustment for multiple comparisons
☐	☒	A full description of the statistical parameters including central tendency (e.g. means) or other basic estimates (e.g. regression coefficient) AND variation (e.g. standard deviation) or associated estimates of uncertainty (e.g. confidence intervals)
☐	☒	For null hypothesis testing, the test statistic (e.g. *F*, *t*, *r*) with confidence intervals, effect sizes, degrees of freedom and *P* value noted<br>*Give P values as exact values whenever suitable.*
☒	☐	For Bayesian analysis, information on the choice of priors and Markov chain Monte Carlo settings
☒	☐	For hierarchical and complex designs, identification of the appropriate level for tests and full reporting of outcomes
☒	☐	Estimates of effect sizes (e.g. Cohen's *d*, Pearson's *r*), indicating how they were calculated

Our web collection on statistics for biologists contains articles on many of the points above.

Software and code

Policy information about availability of computer code

Data collection	Data were collected using BD FACSDiva (v8.0.1).
Data analysis	Flow cytometry data were analyzed by Flowjo 10.6.1. Data was exported into Graphpad prism (version 8.2.0) for statistical analysis. Microscopy analysis was performed using Volocity software (version 6.3.0, PerkinElmer). Paired-end sequencing fastq files were processed using the MiXCR software (v3.0.1).

For manuscripts utilizing custom algorithms or software that are central to the research but not yet described in published literature, software must be made available to editors/reviewers. We strongly encourage code deposition in a community repository (e.g. GitHub). See the Nature Research guidelines for submitting code & software for further information.

Data

Policy information about availability of data

All manuscripts must include a data availability statement. This statement should provide the following information, where applicable:

- Accession codes, unique identifiers, or web links for publicly available datasets
- A list of figures that have associated raw data
- A description of any restrictions on data availability

The TCR sequencing data files can be found on ArrayExpress with accession E-MTAB-8711. The flow cytometry and imaging data supporting the findings are available from the corresponding author upon request. The source data underlying Figures 1A, B, D, F, H, 2A, D, E, 3C, D, 4C, 4, F, 5C, D, E, F, G, 6B, C, D, E, 7A, B, C, D, E, Supplementary Figures 1B, C, D, E, F, G, H, 2B, C, D, 3A, D, F, G, H, 4A, B, C, D, E, 5A, B, C, D, E, F, 6A, B are provided in the Source Data File.

Field-specific reporting

Please select the one below that is the best fit for your research. If you are not sure, read the appropriate sections before making your selection.

☒ Life sciences ☐ Behavioural & social sciences ☐ Ecological, evolutionary & environmental sciences

For a reference copy of the document with all sections, see nature.com/documents/nr-reporting-summary-flat.pdf

Life sciences study design

All studies must disclose on these points even when the disclosure is negative.

Sample size

Between 3-5 mice were used per group within each experiment. This number was based on previous experience with the infection model (Torti et. al. Plos Pathogens 2011, Baumann et.al Plos Pathogens 2019), the availability of the mice, and feasibility of the experiment. No statistical test was used to predetermine sample size.

Data exclusions

1 mouse was excluded from the sequencing analysis because the number of sequencing reads for 1 sample was too low. As we compared two populations in 1 mouse we also excluded the other sample from this mouse because no comparison was possible. Exclusion criteria were not pre-established.

Replication

To verify the reproducibility, experiments were performed at least twice with similar conditions. The majority of experiments was performed three times. When results were not repeated this is stated in the manuscript. All attempts at replication were succesful.

Randomization

Mice were randomly distributed amongst groups at the start of each experiment.

Blinding

Blinding was not performed, due to technical reasons. For in vivo experiments it is required by local authorities to state on the cage cards all handling that is done to the mice. Investigators were not blinded during data collection and analysis.

Reporting for specific materials, systems and methods

We require information from authors about some types of materials, experimental systems and methods used in many studies. Here, indicate whether each material, system or method listed is relevant to your study. If you are not sure if a list item applies to your research, read the appropriate section before selecting a response.

Materials & experimental systems

n/a	Involved in the study
☐	☒ Antibodies
☐	☒ Eukaryotic cell lines
☒	☐ Palaeontology
☐	☒ Animals and other organisms
☒	☐ Human research participants
☒	☐ Clinical data

Methods

n/a	Involved in the study
☒	☐ ChIP-seq
☐	☒ Flow cytometry
☒	☐ MRI-based neuroimaging

Antibodies

Antibodies used

Marker Fluorochrome Manufacturer Catalag number Clone Purpose Dilution Reactivity Validation Manufacturer
B220 Biotin Biolegend 103204 RA3-6B2 Enrichment 1/100 Mouse Staining on C57BL/6 mouse splenocytes
B220 APC BioLegend 103212 RA3-6B Microscopy 1/100 Mouse Staining on C57BL/6 mouse splenocytes
CD4 Biotin Biolegend 100404 GK1.5 Enrichment 1/100 Mouse Staining on C57BL/6 mouse splenocytes
CD8 BV421 BioLegend 100738 53-6.7 Microscopy 1/100 Mouse Staining on C57BL/6 mouse splenocytes
CD8 Percp BioLegend 100732 53-6.7 Flow Cytometry 1/200 Mouse Staining on C57BL/6 mouse splenocytes
CD8 BV510 BioLegend 100752 53-6.7 Flow Cytometry 1/500 Mouse Staining on C57BL/6 mouse splenocytes
CD8 BV605 Biolegend 100744 53.6.7 Flow Cytometry 1/500 Mouse Staining on C57BL/6 mouse splenocytes
CD8 BV570 BioLegend 100739 53-6.7 Flow Cytometry 1/200 Mouse Staining on C57BL/6 mouse splenocytes
CD27 BV421 Biolegend 124223 LG.3A10 Flow Cytometry 1/200 Mouse Staining on C57BL/6 mouse splenocytes
CD39 AlexaFluor 647 BioLegend 143807 Duha59 Flow Cytometry 1/200 Mouse Staining on C57BL/6 mouse splenocytes
CD44 PE BioLegend 103008 IM7 Flow Cytometry 1/500 Mouse Staining on C57BL/6 mouse splenocytes
CD45.1 PE BioLegend 110708 A20 Microscopy 1/200 Mouse Staining on C57BL/6 mouse splenocytes
CD45.1 APC BioLegend 110714 A20 Flow Cytometry 1/200 Mouse Staining on C57BL/6 mouse splenocytes
CD45.1 BV421 BioLegend 110731 A20 Flow Cytometry 1/400 Mouse Staining on C57BL/6 mouse splenocytes
CD45.1 PB BioLegend 110722 A20 Flow Cytometry 1/200 Mouse Staining on C57BL/6 mouse splenocytes
CD45.1 BV510 BioLegend 110741 A20 Flow Cytometry 1/200 Mouse Staining on C57BL/6 mouse splenocytes
CD45.1 BV711 Biolegend 110739 A20 Flow Cytometry 1/300 Mouse Staining on C57BL/6 mouse splenocytes

CD45.2 Percp BioLegend 109825 104 Flow Cytometry 1/200 Mouse Staining on C57BL/6 mouse splenocytes
CD62L Percp BioLegend 104430 MEL-14 Flow Cytometry 1/200 Mouse C57BL/6 mouse bone marrow cells were stained with CD62L
CD62L BUV395 BD 740218 MEL-14 Flow Cytometry 1/200 Mouse Staining on mouse splenocytes
CD90.1 APC eBioscience 17-0900-82 HIS51 Flow Cytometry 1/1000 Mouse The HIS51 antibody has been tested by flow cytometric analysis of rat thymocyte and splenocyte suspensions.
CD127 BV421 BioLegend 135023 A7R34 Flow Cytometry 1/100 Mouse Staining on C57BL/6 mouse splenocytes
CD169 AF647 BioLegend 142408 3D6.112 Microscopy 1/200 Mouse C57BL/6 mouse bone marrow cells were stained
CX3CR1 APC Biolegend 149007 SA011F11 Flow Cytometry 1/200 Mouse Staining on C57BL/6 mouse splenocytes
CXCR5 Biotin Biolegend 145510 L138D7 Flow Cytometry 1/100 Mouse Staining on C57BL/6 mouse splenocytes
IFNγ PECy7 Biolegend 505826 XMG1.2 Flow Cytometry 1/200 Mouse Intracellular staining on PMA + Ionomycin stimulated C57BL/6 splenocytes
IgM BV421 BioLegend 406517 RMM-1 Microscopy 1/100 Mouse Staining on C57BL/6 mouse splenocytes
IL-2 PE BioLegend 503808 JES6-5H4 Flow Cytometry 1/100 Mouse Intracellular staining on PMA + Ionomycin stimulated C57BL/6 splenocytes
KLRG1 PECy7 eBioscience 25-5893-82 2F1 Flow Cytometry 1/200 Mouse This 2F1 antibody has been tested by flow cytometric analysis of mouse splenocytes.
KLRG1 BUV395 BD 740279 2F1 Flow Cytometry 1/200 Mouse Staining on C57BL/6 mouse splenocytes
Lag3 PE BD 552380 C9B7W Flow Cytometry 1/200 Mouse Staining of CD3 activated C57BL/6 splenocytes
Ly108 APC BioLegend 134609 330-AJ Flow Cytometry 1/200 Mouse Staining on C57BL/6 mouse splenocytes
PD-1 PECy7 Biolegend 135215 29F.1A12 Flow Cytometry 1/200 Mouse Con-A stimulated C57BL/6 mouse splenocytes
Streptavidin APC Biolegend 405207 Flow Cytometry 1/500 Each lot of this Streptavidin-APC is quality control tested by immunofluorescent staining with flow cytometric analysis.
Tcf1 PE Cell signaling 14456S C63D9 Flow Cytometry 1/200 Mouse Flow cytometric analysis of Jurkat cells
Tim-3 PE Biolegend 119703 RMT3-23 Flow Cytometry 1/200 Mouse Mouse Tim-3 transfected cells stained with anti-mouse CD366
Va2 PE BioLegend 127808 B20.1 Flow Cytometry 1/500 Mouse Staining on C57BL/6 mouse splenocytes
CD62L n.a. B-Xcell BE0021 MEL-14 In vivo treatment Mouse

Validation

In the table above for each antibody the validation method is indicated in the last column. The validation has been performed by the manufacturer and their statement is used.

Eukaryotic cell lines

Policy information about cell lines

Cell line source(s)

M2-10B4 cells (ATCC # CRL-1972), BHK-21 [C-13] (ATTCC CCL-10)

Authentication

The cell lines were not authenticated in our lab.

Mycoplasma contamination

Cell lines used for virus production were free of Mycoplasma contamination.

Commonly misidentified lines (See ICLAC register)

No commonly misidentified lines were used in this study.

Palaeontology

Specimen provenance

Provide provenance information for specimens and describe permits that were obtained for the work (including the name of the issuing authority, the date of issue, and any identifying information).

Specimen deposition

Indicate where the specimens have been deposited to permit free access by other researchers.

Dating methods

If new dates are provided, describe how they were obtained (e.g. collection, storage, sample pretreatment and measurement), where they were obtained (i.e. lab name), the calibration program and the protocol for quality assurance OR state that no new dates are provided.

☐ Tick this box to confirm that the raw and calibrated dates are available in the paper or in Supplementary Information.

Animals and other organisms

Policy information about studies involving animals; ARRIVE guidelines recommended for reporting animal research

Laboratory animals

C57BL/6J were purchased from Janvier Elevage and were used as WT mice. CD45.1, CD45.1 Maxi, Tcf7GFP , CD45.1 Tcf7 GFP P14, CD45.1 Tcf7 Maxi, CD45.2 Tcf7DTR-GFP P14 , CD45.1Thy1.1 IL12Rβ2-/- P14, Thy1.1 IFNAR-/- P14, CD45.1 Mini and CD45.1 CCR7-/- Mini mice all on a C57Bl/6 background are also used in this study. Both male or female mice were used and they were sex- and age- matched within each experiment. Mice were between 7-12 weeks of age at the start of each experiment.

Wild animals	The study did not involve wild animals.
Field-collected samples	The study did not involve samples collected from the field.
Ethics oversight	This study was conducted in accordance to the guidelines of the animal experimentation law (SR 455.163; TVV) of the Swiss Federal Government. The protocol was approved by Cantonal Veterinary Office of the canton Zurich, Switzerland (Permit number 146/2014, 114/2017 and 115/2017).

Note that full information on the approval of the study protocol must also be provided in the manuscript.

Human research participants

Policy information about studies involving human research participants

Population characteristics	*Describe the covariate-relevant population characteristics of the human research participants (e.g. age, gender, genotypic information, past and current diagnosis and treatment categories). If you filled out the behavioural & social sciences study design questions and have nothing to add here, write "See above."*
Recruitment	*Describe how participants were recruited. Outline any potential self-selection bias or other biases that may be present and how these are likely to impact results.*
Ethics oversight	*Identify the organization(s) that approved the study protocol.*

Note that full information on the approval of the study protocol must also be provided in the manuscript.

Clinical data

Policy information about clinical studies

All manuscripts should comply with the ICMJEguidelines for publication of clinical research and a completedCONSORT checklist must be included with all submissions.

Clinical trial registration	*Provide the trial registration number from ClinicalTrials.gov or an equivalent agency.*
Study protocol	*Note where the full trial protocol can be accessed OR if not available, explain why.*
Data collection	*Describe the settings and locales of data collection, noting the time periods of recruitment and data collection.*
Outcomes	*Describe how you pre-defined primary and secondary outcome measures and how you assessed these measures.*

ChIP-seq

Data deposition

☐ Confirm that both raw and final processed data have been deposited in a public database such as GEO.

☐ Confirm that you have deposited or provided access to graph files (e.g. BED files) for the called peaks.

Data access links<br>*May remain private before publication.*	*For "Initial submission" or "Revised version" documents, provide reviewer access links. For your "Final submission" document, provide a link to the deposited data.*
Files in database submission	*Provide a list of all files available in the database submission.*
Genome browser session<br>(e.g. UCSC)	*Provide a link to an anonymized genome browser session for "Initial submission" and "Revised version" documents only, to enable peer review. Write "no longer applicable" for "Final submission" documents.*

Methodology

Replicates	*Describe the experimental replicates, specifying number, type and replicate agreement.*
Sequencing depth	*Describe the sequencing depth for each experiment, providing the total number of reads, uniquely mapped reads, length of reads and whether they were paired- or single-end.*
Antibodies	*Describe the antibodies used for the ChIP-seq experiments; as applicable, provide supplier name, catalog number, clone name, and lot number.*
Peak calling parameters	*Specify the command line program and parameters used for read mapping and peak calling, including the ChIP, control and index files used.*
Data quality	*Describe the methods used to ensure data quality in full detail, including how many peaks are at FDR 5% and above 5-fold enrichment.*

Software

Describe the software used to collect and analyze the ChIP-seq data. For custom code that has been deposited into a community repository, provide accession details.

Flow Cytometry

Plots

Confirm that:

- [x] The axis labels state the marker and fluorochrome used (e.g. CD4-FITC).
- [x] The axis scales are clearly visible. Include numbers along axes only for bottom left plot of group (a 'group' is an analysis of identical markers).
- [x] All plots are contour plots with outliers or pseudocolor plots.
- [x] A numerical value for number of cells or percentage (with statistics) is provided.

Methodology

Sample preparation

Single cell suspensions were prepared from spleen and lymph nodes by meshing the tissue through a 70 µM cell strainer. Erythrocytes were lysed using a hypotonic ammonium-chloride-potassium buffer for 1 minute. To collect lungs, mice were perfused with PBS to remove all blood associated cells. To prepare a single cell suspension from the lungs, the tissue was cut into small pieces, subsequently incubated with Collegenase I and DNAse I for 45 minutes, followed by a 30% percoll gradient. Cells were incubated with fluorescently conjugated antibodies for 30 minutes at 4ºC. Dead cells were excluded using a LIVE/DEAD fixable NEAR-IR staining. For intracellular staining of Tcf1, the FoxP3 kit (invitrogen) was used according to manufacterer's protocol. For intracellular cytokine staining, sorted Tcf1+ or Tcf1- P14 T cells were in vitro restimulated for 5 hours with 1 µg/ml GP33-41 peptide in the presence of 2 µg/ml Brefeldin A. After stimulation, the cell surface was stained for 30 minutes at 4ºC, after which the cells were washed and fixed overnight with 0.5% PFA. The following day, cells were washed with perm/wash buffer (eBioscience) and cytokines were stained intracellular for 30 minutes at 4ºC. The fluorescently conjugated antibodies were diluted in perm/wash buffer.

Instrument

Multi-parametric flow cytometric analysis was performed using LSRII and Fortessa flow cytometer (BD Biosciences)

Software

Flow cytometry data were analyzed by Flowjo 10.6.1.

Cell population abundance

We enriched for TCR transgenic CD8 T cells (P14, MAXI), using the mojosort CD8 T cell isolation kit according to manufacturer's protocol (Biolegend). Purity was always assessed by flow cytometry before cells were used. Post-sort fractions were also acquired directly after sorting to check the purity. This was always above 98%.

Gating strategy

For all samples the following gating strategy was used, exclusion of doublets (FSC-H/FSC-A), leukocytes (SSC-A/FSC-A), live cells (Near IR/FSC-A) .
For tetramer staining, cells were gated on CD8, and subsequently CD44 was plotted versus tetramer.
For transfer of congenically marked TCR transgenic CD8 T cells, cells were gated for CD8 and for the congenic marker.
For intracellular cytokine staining, no Near IR staining was included. Cells were gated on CD8 and CD45.1, and subsequently for the cytokines.

- [x] Tick this box to confirm that a figure exemplifying the gating strategy is provided in the Supplementary Information.

Magnetic resonance imaging

Experimental design

Design type

Indicate task or resting state; event-related or block design.

Design specifications

Specify the number of blocks, trials or experimental units per session and/or subject, and specify the length of each trial or block (if trials are blocked) and interval between trials.

Behavioral performance measures

State number and/or type of variables recorded (e.g. correct button press, response time) and what statistics were used to establish that the subjects were performing the task as expected (e.g. mean, range, and/or standard deviation across subjects).

Acquisition

Imaging type(s)

Specify: functional, structural, diffusion, perfusion.

Field strength

Specify in Tesla

Sequence & imaging parameters

Specify the pulse sequence type (gradient echo, spin echo, etc.), imaging type (EPI, spiral, etc.), field of view, matrix size, slice thickness, orientation and TE/TR/flip angle.

Area of acquisition

State whether a whole brain scan was used OR define the area of acquisition, describing how the region was determined.

Diffusion MRI ☐ Used ☐ Not used

Preprocessing

Preprocessing software	*Provide detail on software version and revision number and on specific parameters (model/functions, brain extraction, segmentation, smoothing kernel size, etc.).*
Normalization	*If data were normalized/standardized, describe the approach(es): specify linear or non-linear and define image types used for transformation OR indicate that data were not normalized and explain rationale for lack of normalization.*
Normalization template	*Describe the template used for normalization/transformation, specifying subject space or group standardized space (e.g. original Talairach, MNI305, ICBM152) OR indicate that the data were not normalized.*
Noise and artifact removal	*Describe your procedure(s) for artifact and structured noise removal, specifying motion parameters, tissue signals and physiological signals (heart rate, respiration).*
Volume censoring	*Define your software and/or method and criteria for volume censoring, and state the extent of such censoring.*

Statistical modeling & inference

Model type and settings	*Specify type (mass univariate, multivariate, RSA, predictive, etc.) and describe essential details of the model at the first and second levels (e.g. fixed, random or mixed effects; drift or auto-correlation).*
Effect(s) tested	*Define precise effect in terms of the task or stimulus conditions instead of psychological concepts and indicate whether ANOVA or factorial designs were used.*

Specify type of analysis: ☐ Whole brain ☐ ROI-based ☐ Both

Statistic type for inference<br>(See Eklund et al. 2016)	*Specify voxel-wise or cluster-wise and report all relevant parameters for cluster-wise methods.*
Correction	*Describe the type of correction and how it is obtained for multiple comparisons (e.g. FWE, FDR, permutation or Monte Carlo).*

Models & analysis

n/a	Involved in the study
☐	☐ Functional and/or effective connectivity
☐	☐ Graph analysis
☐	☐ Multivariate modeling or predictive analysis

Functional and/or effective connectivity	*Report the measures of dependence used and the model details (e.g. Pearson correlation, partial correlation, mutual information).*
Graph analysis	*Report the dependent variable and connectivity measure, specifying weighted graph or binarized graph, subject- or group-level, and the global and/or node summaries used (e.g. clustering coefficient, efficiency, etc.).*
Multivariate modeling and predictive analysis	*Specify independent variables, features extraction and dimension reduction, model, training and evaluation metrics.*
